# A six-herb Chinese medicine composition ointment as a promising candidate for treatment of hypertrophic scars

**DOI:** 10.1016/j.chmed.2020.12.007

**Published:** 2020-12-29

**Authors:** Zu-hua Wang, Xue-yan Sun, Jiao-jiao Zhang, Francesca Giampieri, Cheng-ju Jiang, Ting-ting Feng, Zhi-wei Wang, Rong-yi Chen, Maurizio Battino, Ying Zhou

**Affiliations:** a College of Pharmaceutical Sciences, Guizhou University of Traditional Chinese Medicine, Guiyang 550025, China; b Nano-drug Technology Research Center of Guizhou University of Traditional Chinese Medicine, Guiyang 550025, China; c Department of Clinical Sciences, Faculty of Medicine, Università Politecnica delle Marche, Ancona 60131, Italy; d Department of Biochemistry, Faculty of Sciences, King Abdulaziz University, Jeddah 21589, Saudi Arabia.; e QianDongNan Vocational & Technical College for Nationalities, Kaili 556000, China; f School of Medicine, Southern Medical University, Guangzhou 510091, China; g International Research Center for Food Nutrition and Safety, Jiangsu University, Zhenjiang 212013, China

**Keywords:** anti-hypertrophic scar effect, Chinese medicine, hypertrophic scars, ointment, orthogonal design

## Abstract

**Objective:**

To study the anti-hypertrophic scar effect of the six-herb Chinese medicine composition (SCMC) ointment on the rabbit ear hypertrophic scar models.

**Methods:**

The optimal formulation of SCMC ointment matrix was screened by the orthogonal designs and a series of evaluation tests. The SCMC ointment was prepared through emulsifying method. The rabbit ear hypertrophic scar models were established and used to investigate the anti-hypertrophic scar effect of SCMC ointment.

**Results:**

Our results demonstrated that all the quality control indications of the SCMC ointment met the requirements. *A*nti-hypertrophic scar activity results showed that all the rabbit ear scar tissues appeared different degrees of shrink and fading, and took an unobvious but palpable shift from hard to soft texture with the low, middle and high concentration SCMC ointments treatments *in vivo*. Additionally, on 21st day the scar area and thickness in different concentrations of SCMC ointment groups were significantly reduced than control group, in a concentration-dependent manner. The immunohistochemical results also indicated that the SCMC ointment had good anti-hypertrophic scar properties and could inhibit hypertrophic scar formation.

**Conclusion:**

The SCMC ointment could improve the blood circulation condition of hypertrophic scar tissues. Our research has demonstrated the Chinese medicine composition ointment with good anti-hypertrophic scar properties that could be used to treat hypertrophic scars. Meanwhile, it provides a theoretical basis for further clinical application.

## 1 Introduction

Keloid scarring is a pathophysiological phenomenon of continuous growing beyond the boundary of the original wound margin, which commonly occurs in pigmented skin post cutaneous injury (Kant et al., 2018, Lebeko et al., 2019, Supp, 2019). As a kind of tissue fibroproliferative disorders, hypertrophic scars and keloids could be caused by certain physiological and pathological factors (i.e. burns, trauma, inflammation and abnormal wound healing) (Aarabi et al., 2007, Hawkins et al., 2018, Khatib et al., 2018), which significantly impair the quality of life of patients. So far, hypertrophic scar has still been a troublesome clinical problem, whose mechanisms are still obscure. Previous studies have suggested that this is due to a combination of biochemical factors, skin tension, endocrinologic factors, and genetic factors (Brissett & Sherris, 2001). In pathological aspect, an increase in fibroblast proliferation and collagen expression is one of the most eminent characters of hypertrophic scars and keloids (Zuo et al., 2018).

There are many ways for the prevention and treatment of hypertrophic scars, which are generally divided into operative therapy, medicines, physiotherapy and comprehensive treatments. Usually, large area of keloidal scar can be avoided during connective tissue repair, such as dermatoplasty. However, dermatoplasty is not suitable to treat the small areas of hypertrophic scars and keloids. Except the personal risks, it is very expensive and hard to implement. Meanwhile, the surgical procedures could increase the incidence of scarring. With the development of medicine, absorbable suture had been widely used in surgical operation that was expected to reduce the scarring left, especially on the face, which cannot satisfy the beauty lover’s need for appearance (McCue & Kalliainen, 2018). Autologous fat grafting has been used in the treatment of scar tissue and scar-related conditions that is the time-honored manner (Negenborn, Groen, Smit, Niessen, & Mullender, 2016). Unfortunately, autologous fat grafting is also not suitable for treating the hypertrophic scars and keloids. Among all the methods, no-operative therapy is widely used to treat hypertrophic scars and keloids. Laser (Patel et al., 2019), pressure (Li et al., 2018), radiotherapy (Renz et al., 2018), medicine (Waibel, Wulkan, Rudnick, & Daoud, 2019), traditional Chinese medicine (Xie et al., 2009) and physiotherapy (Karimi, Mobayen, & Alijanpour, 2013) are the most common therapeutic strategies of no-operative therapies. Increasing medical researchers have paid attention to no-operative therapies of the hypertrophic scars and keloids and made more progress in recent years. With radiation therapy as example, a long-term follow-up study showed that radiation therapy following excision of keloids has been shown to decrease the recurrence rate by about 50% (Kovalic & Perez, 1989).

Traditional Chinese medicine has always been recognized as a holistic unity from the human physiopathology, which is undoubtedly more in line with the needs of human health (Sun et al., 2013). In recent years, traditional Chinese medicine attracts a lot of attentions and even has become a very active field due to its wide variety of pharmaceutically active compounds and unexpected effects on various diseases, including the hypertrophic scars and keloids (He et al., 2012). Many traditional Chinese medicines have the potential effect in promoting blood circulation and strengthening the capacity of human metabolism. Therefore, the compounds with antioxidation, anti-proliferative, and anti-inflammatory activities are likely to be proposed for anti-keloid treatment, which are wildly distributed in traditional Chinese medicine, i.e. tanshinone II-A (*Salvia miltiorrhiza* Bge., Danshen in Chinese), matrine (*Sophora flavescens* Ait., Kushen in Chinese), gallotannin (*Galla Chinensis*, Wubeizi in Chinese), ligustrazine (*Ligusticum wallichii* Franch., Chuanxiong in Chinese) and so on (Djakpo and Yao, 2010, Ferrandiz and Devesa, 2008, Ho et al., 2009, Zhang et al., 2009). Based on the theory of traditional Chinese medicine and therapeutic method of diseases, Chinese medicines were screened for their potential anti-scarring effect according to the principle of activating blood and resolving stasis, softening hardness and dissipating mass (also called “Huo-Xue-Hua-Yu, Ruan-Jian-San-Jie”, a concept of traditional Chinese medical theory) (Chen and Chen, 2015, Xu et al., 1989), such as that *S. miltiorrhiza, L. wallichii*, and *Carthamus tinctorius* L. can activate the blood circulation and eliminate stasis (Chen et al., 2014, Guo et al., 2013, Wang et al., 2019), *Galla Chinensis* and *S. flavescens* have good effect of Ruan-Jian-San-Jie and inhibitory effects on fibroblast cells (Yang et al., 2002, Yulu et al., 1994), *Brucea javanica* (L.) Merr. (Yadanzi in Chinese) *has* effect of corroding excrescence (Jin et al., 2015). Under the guidance of the theory and principle, we finally selected *S. miltiorrhiza, S. flavescens, Galla Chinensis, L. wallichii, B. javanica* (Lee et al., 1979) and *C. tinctorius* (Tse, 2003) as an optimized Chinese medicine compound recipe for the further treatment of the hypertrophic scars and keloids.

Ointments are considered as the simplest and convenient medication dosage form with high compliance and flexibility (Kandhare, Alam, Patil, Sinha, & Bodhankar, 2016), which enjoys a good quality in adhesion, spread stretch ability and portability. Owing to these advantages, ointments are widely employed as the media for transdermal drug or cosmetic delivery and have potential application prospects on skin-based diseases (Lepselter, Britva, Karni, & Issa, 2017). According to the results of the current study, a six-herb Chinese medicine-based composition (SCMC) ointment including above Chinese medicine extracts was evaluated as a viable treatment option for scar management. In this work, stearic acid, glycerol monostearate and sodium dodecyl sulfate were selected as investigation factors (each factor with three levels) and designed by L9 (33) orthogonal arrays to obtain the optimal ointment bases formulation. Additionally, in order to investigate the anti-hypertrophic scar effect of SCMC ointment, we successfully established the rabbit ear hypertrophic scar models for further evaluation the efficacy of the compound traditional Chinese medicinal ointment treatment by carrying a topical therapy onto the surface of this scar models.

**Fig. 1:**
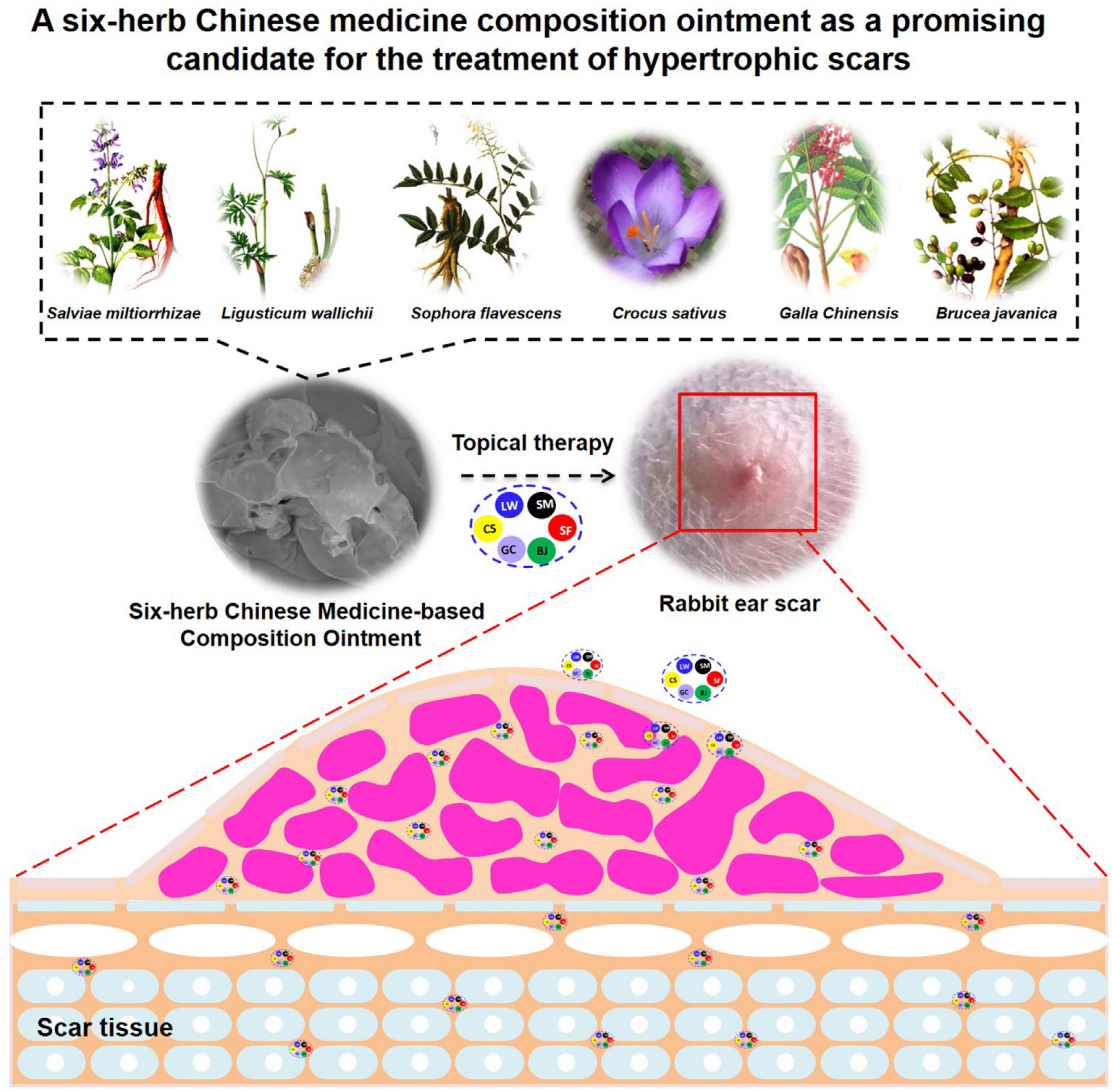
Schematic illustration of topical therapy with Six-herb Chinese Medicine-based Composition (SCMC) Ointment on hypertrophic Scar.

Fig. 1 showed the schematic illustration of topical therapy with SCMC ointment on hypertrophic scar. As shown in Fig. 1, the extracts of six Chinese medicines were applied to prepare the SCMC ointment, including *S. miltiorrhizae, L. wallichii, S. flavescens, Crocus sativus* L. (Xihonghua in Chinese)*, Galla Chinensis and B. javanica*. To further investigate the potential therapy effect of SCMC ointment on the hypertrophic scars and keloids, a topical therapy was carried out in the rabbit ear-based hypertrophic scar models. Therefore, this work is expected to provide a reliable, safe and convenient strategy for the treatment of hypertrophic scars and keloids.

## 2 Material and methods

### 2.1 Materials

*S. miltiorrhizae* extract (containing 96% tanshinone II-A), *S. flavescens* extract (containing 98% matrine) and *Galla Chinensis* extract (containing 98% gallotannin) were purchased from Xian Tianxiang Biological Engineering Co., Ltd. (Xian, China), *B. javanica* extract (oleic acid at a concentration of 0.14 g/mL), *L. wallichii* extract (containing 98% tetramethylpyrazine) and *C. sativus* extract (containing 2% hydroxy safflower yellow A) were purchased from Jianshengda Spice Oil Co., Ltd (Jiangxi, China)., Shanxi Guanchen Biotechnology Co., Ltd., (Shanxi, China) and Shanxi Tianxingjian Bio-chemical Technology Co., Ltd. (Shanxi, China), respectively, and the above reference substances were purchased from Aladdin (Shanghai, China). Stearic acid, white vaseline and glyceryl monostearate were provided by Shanghai Macklin Biochemical Technology Co., Ltd (Shanghai, China). Sodium dodecyl sulfate (SDS) was purchased from Shanghai Titan Technology Co., Ltd (Shanghai, China). Ethyl4-hydroxybenzoate was purchased from Saan Chemical Technology (Shanghai) Co., Ltd (Shanghai, China). Azone was purchased from Zhejiang Tianhong Biotechnology Co., Ltd (Zhejiang, China). Glycerin was purchased from Chongqing Wansheng Chuandong Chemical Co., Ltd (Chongqing, China). Van Gieson (VG) was purchased from Bomei Biotechnology Co., Ltd (Hefei, China). Neutral balsam was purchased from Sinopharm Chemical Reagent Co., Ltd (Shanghai, China). All other solvents were of analytical or chromatographic grade.

### 2.2 Optimization of ointment bases formulation

It is the oil-in-water (o/w) emulsion-type ointment base that exhibits excellent properties such as good unctuousness and spreadability, so we select it as a vector of the drug delivery. According to the preliminary ointment bases formulation design, the stearic acid, glyceryl monostearate, white vaseline and azone were selected as the oil phase, the distilled water and glycerin as the water phase, sodium dodecyl sulfate (SDS) as the emulsifying agent and ethyl4-hydroxybenzoate as the preservative. The emulsifying temperature was confirmed at 85 °C. In order to obtain the optimal ointment bases formulation, orthogonal design and a series of verified experiments were carried out. During the experiment process, we determined the test indicators and related scoring standards. The test indicators of ointment bases formulation included the appearance, centrifugal stability, heat/cold resistant properties test, pH range and skin stimulation, then each test was scored according to the scoring standards as shown in Table 1.

**Table 1:** Scoring standards of each test.

Levels	Excellent(20)	Good(10)	Qualified(4)	Unqualified(0)
Appearance	good unctuousness and spreadability	rough, good spreadability	qualitative hard, bad spreadability	oil water dissociation
Centrifugal stability	unchanged	slight layering	oil droplets precipitated	demulsification
Heat/cold resistant properties	unchanged	harden or soften	−	demulsification
pH range	pH 6–8.3	−	−	other pH
Skin stimulation	none	mild erythema or edema	moderate erythema or edema	severe erythema or edema

In addition, stearic acid, glycerol monostearate and sodium dodecyl sulfate were selected as investigation factors (each factor with three levels) and designed by L9 (33) orthogonal arrays (Chua Abdullah, Abdollah, Amiruddin, Tamaldin, & Mat Nuri, 2014), which optimal combination effects were also examined in the orthogonal test according to the scoring. The analysis of variance was carried out by SPSS software in order to optimize the ointment bases formulation. The level of factors was shown in Table 2. Finally, the optimal ointment bases formulation was determined through the above analysis and further formulation verification.

**Table 2:** Factors and levels of orthogonal test.

Levels	A: Stearic acid/g	B: Glycerol monostearate/g	C: Sodium dodecyl sulfate/g
1	10	3	0.5
2	17.5	4	1.25
3	25	5	2

### 2.3 Preparation of six-herb Chinese medicine-based composition ointment

The six-herb Chinese medicine-based composition (SCMC) ointment was prepared according to the above optimal matrix formulation by the emulsification method. Briefly, 52.5 g of stearic acid, 12 g of glyceryl monostearate, 24 g of white vaseline and 12 g of azone were subjected to heat/dissolved at 85 °C as an oil phase. 37.5 g of sodium dodecyl sulfate (SDS), 0.45 g of ethyl4-hydroxybenzoate and 40.8 g of glycerin were subjected to heat/dissolved at 75 °C as a water phase. A total of 0.315 g of *S. miltiorrhizae* extract (containing 96% tanshinone II-A), 0.306 g of *S. flavescens* extract (containing 98% matrine) and 13 μL of *B. javanica* extract (oleic acid at a concentration of 0.14 g/mL) were suspended in the oil phase and heated with frequent agitation to completely dissolve the extracts and distribute into flasks as an extracts-based oil phase. A total of 0.161 g of *Galla Chinensis* extract (containing 98% Gallotannin), 0.316 g of *L. wallichii* extract (containing 98% Tetramethylpyrazine) and 0.12 g of *C*. *sativus* extract (containing 2% Hydroxy safflower yellow A) were suspended in the water phase and heated with frequent agitation to completely dissolve the extracts and distribute into beaker as an extracts-based water phase. Then, the extracts-based water phase was poured into the extracts-based oil phase at 85 °C, mixed by swirling until water/oil systems were thoroughly combined. After adding a certain amount of water into the water/oil system to the total weight of 300 g, the mixture was stirred continuously at the same temperature for 30 min until the water/oil phase system was fully homogeneous and well-mixed. After that, the compound system was cooled at room temperature when the stirring was stopped. Finally, the low concentration SCMC ointment was successfully prepared. Simultaneously, the composition ointments with medium and high concentration were also prepared by adjusting the usage amount of the anti-hypertrophic activity compositions to double and triple. The shapes and texture of low, medium and high concentration SCMC ointment were observed under inverted microscope (Olympus IX70, Olympus Optical Co., Tokyo, Japan).

### 2.4 Determination of ingredient composition of SCMC ointment

Ingredient composition of SCMC ointment was simultaneously analyzed by an ultra high-pressure liquid chromatography (UHPLC). The analytical system used was Agilent UHPLC 1290 (Agilent technologies, USA). The column was Agilent ZORBAX Eclipse Plus C_18_, 100 mm × 2.1 mm, 1.8 µm column. The mobile phase was 0.2% phosphoric acid (H_3_PO_4_) (A) and acetonitrile (B), gradient elution: 0–10 min, 98% (A); 10–60 min, 95% (A); 60–70 min, 5% (A); above 70 min, 2% (A). The detection wavelength was set at 270 nm, and injected volume was 5 μL, volume Flowrate was 1 mL/min, column temperature was 30 °C.

### 2.5 Establishment and histological evaluation of rabbit ear-based hypertrophic scar models

The rabbit ear-based hypertrophic scar models were established by a surgical trauma method. All animal studies were carried out under Institutional Animal Care and Use Committee-approved protocols of Guizhou University of Traditional Chinese Medicine (No. GZY20190002, Approval Date Sep 11, 2019). Briefly, healthy adult New Zealand rabbits were anaesthetized with ether and fixed on the special surgical stent. The ear skin of rabbit was sterilized using 0.5% (ratio of mass to volume) iodophor and sought out the skin tissue with few blood vessels distribution. After that, four incisions were made with a scalpel, two up the horizontal and two vertical the ear, like a square. Then, the ear skin within the square was further stripped using a needle knife to obtain the regular square shape (1 cm × 1 cm) rabbit ear-based hypertrophic scar models. Above operation, we paid attention to action to ease, even, could not overexert, so as to avoid damage the rabbit ear during the experiment. Finally, the right amount of 5% iodophors onto rabbit ear wound was dabbed with a cotton swab (three times a day for three consecutive days) to avoid the wound infection. The hypertrophic scar growth of rabbit ear was observed everyday. After 21 d, the rabbits were sacrificed and the healthy and scar ear skins tissues were collected for H&E staining.

### 2.6 *In vivo* anti-hypertrophic scar effect studies

The *in vivo* anti-hypertrophic scar activity of SCMC ointment was investigated in rabbit ear-based hypertrophic scar models. When all rabbit ear-based hypertrophic scar area reached the acceptable sizes (about 15 d, approximately 0.5–0.6 cm^2^) or the thickness (about 15 d, approximately 0.2–0.3 cm), the rabbits were treated by topical smearing on the hypertrophic scar models with different concentrations of SCMC ointment. Rabbits ear bearing hypertrophic scars were randomly allocated into four groups, including saline (*n* = 3, 30 mg saline/kg body weight), low concentration SCMC ointment (*n* = 3, 30 mg SCMC ointment/kg body weight), medium concentration SCMC ointment (*n* = 3, 30 mg SCMC ointment/kg body weight) and high concentration SCMC ointment (*n* = 3, 30 mg SCMC ointment/kg body weight) groups. All rabbits were smeared for a total three times on each day until 21 d. The body weight of each rabbit was monitored at 0, 14th and 21th days. The anti-hypertrophic scar activity of SCMC ointment was determined by measuring the width, length and thickness of the scars after treatment with a vernier calliper.

### 2.7 *In vivo* immunohistochemistry analysis

Van Gieson's Stain is a mixture of picric acid and acid fuchsin. It is the simplest method of differential staining of collagen and other connective tissue. Therefore, Van Gieson was used to evaluate the increase of collagen in diseases. In this study, the specimens of rabbit ear hypertrophic scar tissue were further stained with hematoxylin and Van Gieson (VG) to evaluate the anti-hypertrophic scar effect of SCMC ointment. Briefly, the rabbits were killed by direct injection of air into rabbit central ear artery when the experiment ended. The scar tissues of rabbits were taken out by the surgical scissors. The specimens were fixed in 4% methanal solution for 24 h, and ensured that the fixed sections were adequately embedded in paraffin, then cut tissue sections to 4–5 µm. The paraffin slices were dewaxed in xylene and stained with hematoxylin for 10–20 min. After that, the paraffin slices were washed with the running water for 1–3 min and dealed with hydrochloric acid-ethanol solution for 5–10 s. Washed the slices with water again and put them into warm distilled water (50 °C) until the blue was appeared. After washing with water for 1–3 min again, the slices were put into 85% alcohol for 3–5 min and stained with Van Gieson's picrofuchsin for 1–2 min. Then, the slices were washed with water for 3–4 s, dehydrated in a gradient ethanol series (70%, 80% and 90%), vitrified by dimethylbenzene and mounted in neutral balsam. Finally, the slices were observed at 40× or higher magnification using a digital microscope (BA400 Digital, Mike Audi Industrial Group Co., Ltd. Xiamen, China).

### 2.8 Vascular supply and distribution of hypertrophic scar skin

The vascular changes could be the primary controlling factor in scar maturation (Page, Robertson, & Pettigrew, 1983). Therefore, it has the important significance to investigate the vascular supply and distribution of the hypertrophic scar skin for evaluating the anti-hypertrophic scar effect of SCMC ointment. At the end of the experiment (22 d), the rabbits were anesthetized using ether and fixed on an operating table. Then the vascular supply and distribution of the hypertrophic scar skin were observed by a microcirculation blood flow visualizer (XW880, Jianneng optical instrument Co., Ltd).

### 2.9 Statistical analysis

The statistical significance of the differences between groups was assessed using the Student’s *t*-test at the significance level of *P* < 0.05 for each paired experiment.

## 3 Results

### 3.1 Optimization of ointment bases formulation

Orthogonal experiment is a method that analyzes multiple factors using orthogonal tables, widely used for screening the optimum prescription and preparation technology of pharmaceutical preparations. In this study, the optimal combination of stearic acid, glycerol monostearate and sodium dodecyl sulfate was systematically investigated by orthogonal design method. As shown in Table 3, Table 4, the optimal combination of ointment bases formulation was A_2_B_2_C_2_ according to the variance analysis. The content of stearic acid, glycerol monostearate and sodium dodecyl sulfate respectively was 17.5%, 4% and 1.25%. In addition, among all the factors investigated above, the priority sequence of affecting the stability of ointment bases was C > B > A. The blank ointment prepared according to the optimal bases formulation showed the good properties in appearance, centrifugal stability, heat/cold resistant properties, pH range and skin stimulation, which basically met the requirements of ointment preparation.

**Table 3:** Results of orthogonal test on formulation.

Test numbers	Factors			
	A	B	C	Comprehensive scores
1	1	1	1	40
2	2	2	1	74
3	3	3	1	48
4	2	2	2	80
5	2	1	2	60
6	1	3	2	64
7	1	2	3	44
8	2	3	3	54
9	3	1	3	54
K1	148	154	162	
k2	188	198	204	
K3	182	166	152	
k1	49.333	51.333	54.000	
k2	62.667	66.000	68.000	
k3	60.667	55.333	50.667	
R	13.333	14.667	17.333	
Sequence	C > B > A			
Optimal levels	A_2_B_2_C_2_			
Optimal combination	C_2_B_2_A_2_

**Table 4:** Variance analysis of orthogonal experiment.

Factors	Sum of square deviations	Degree of freedom	Mean square	*F* value	*P* value
Stearic acid	310.221	2	155.111	1.159	0.463
Glycerol monostearate	344.889	2	172.444	1.289	0.436
Sodium dodecyl sulfate	507.556	2	253.778	1.897	0.354
Error	267.555	2			

Critical value *F_0.05_* (2,2) = 19.00

The final formulation of ointment bases was composed of stearic acid 17.5%, glycerol monostearate 4% and sodium dodecyl sulfate 1.25%, ethyl4-hydroxybenzoate 0.15%, azone 4%, white Vaseline 8% and glycerin 13.6%.

### 3.2 Preparation of SCMC ointment

**Fig. 2:**
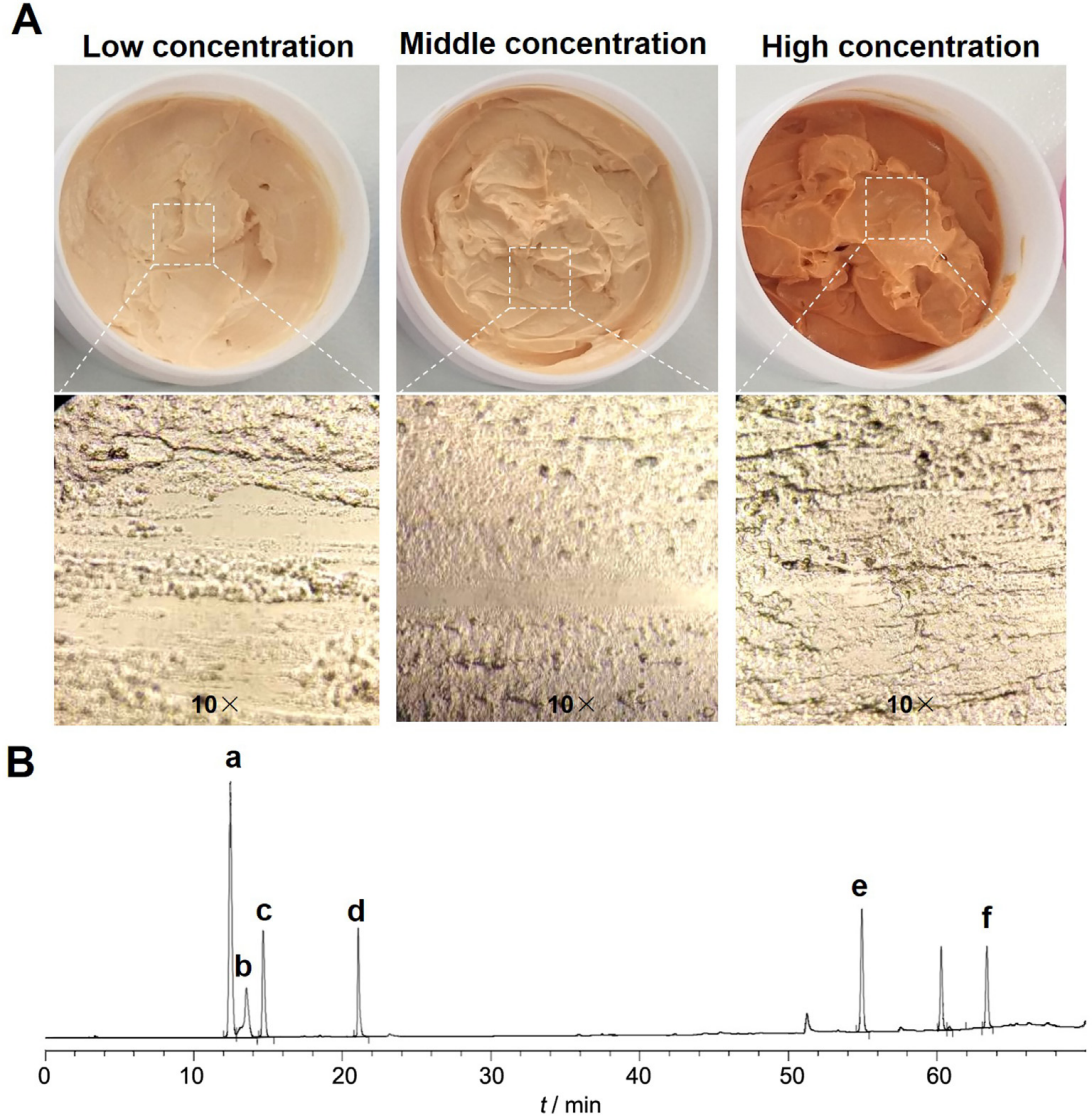
Representative photographs and microscope images of SCMC ointments with low, middle and high concentrations (A); Chromatographic characterization of ingredient composition in SCMC ointment by UHPLC (B), from a to f on the chromatogram are, in order, gallotannin, matrine, ligustrazine, hydroxysafflor yellow A, tanshinone II-A and oleic acid.

Based on the optimization of the ointment bases formulation, the SCMC ointments with different drug concentrations (low, middle and high) were successfully prepared. As shown in Fig. 2A, the paste colors of the low, middle and high concentration SCMC ointments were orange-and-white, orange and orange-red, respectively, highlighting that the SCMC ointment color was related to the content of the Chinese medicine extracts even if it did not affect the good spreadable properties of the ointment. Therefore, all the SCMC ointments showed good appearance, soft and smooth tactility, easy to coat and no odor distribution. The observations by optical microscope also demonstrated that the SCMC ointments with different concentrations had exhibited fine texture. The further experiment results also suggested that all the quality control indications of the SCMC ointments with three concentrations (low, middle and high), including appearance, centrifugal stability, heat/cold resistant properties, pH range and skin stimulation reached the requirements (Table 5). In addition, the result also showed that six antiscaring active components could be simultaneously examined in the SCMC ointment by UHPLC. As shown in Fig. 2B, six main active components were gallotannin, matrine, ligustrazine, hydroxysafflor yellow A, tanshinone II-A and oleic acid.

**Table 5:** Quality score results of SCMC ointments.

Test names	Appearance	Centrifugal stability	Heat/cold resistant properties	pH range	Skin stimulation	Scores
Low concentration	20	20	10	20	20	90
Middle concentration	20	20	10	20	20	90
High concentration	20	20	10	20	10	80

### 3.3 Establishment of rabbit ear-based hypertrophic scar models

**Fig. 3:**
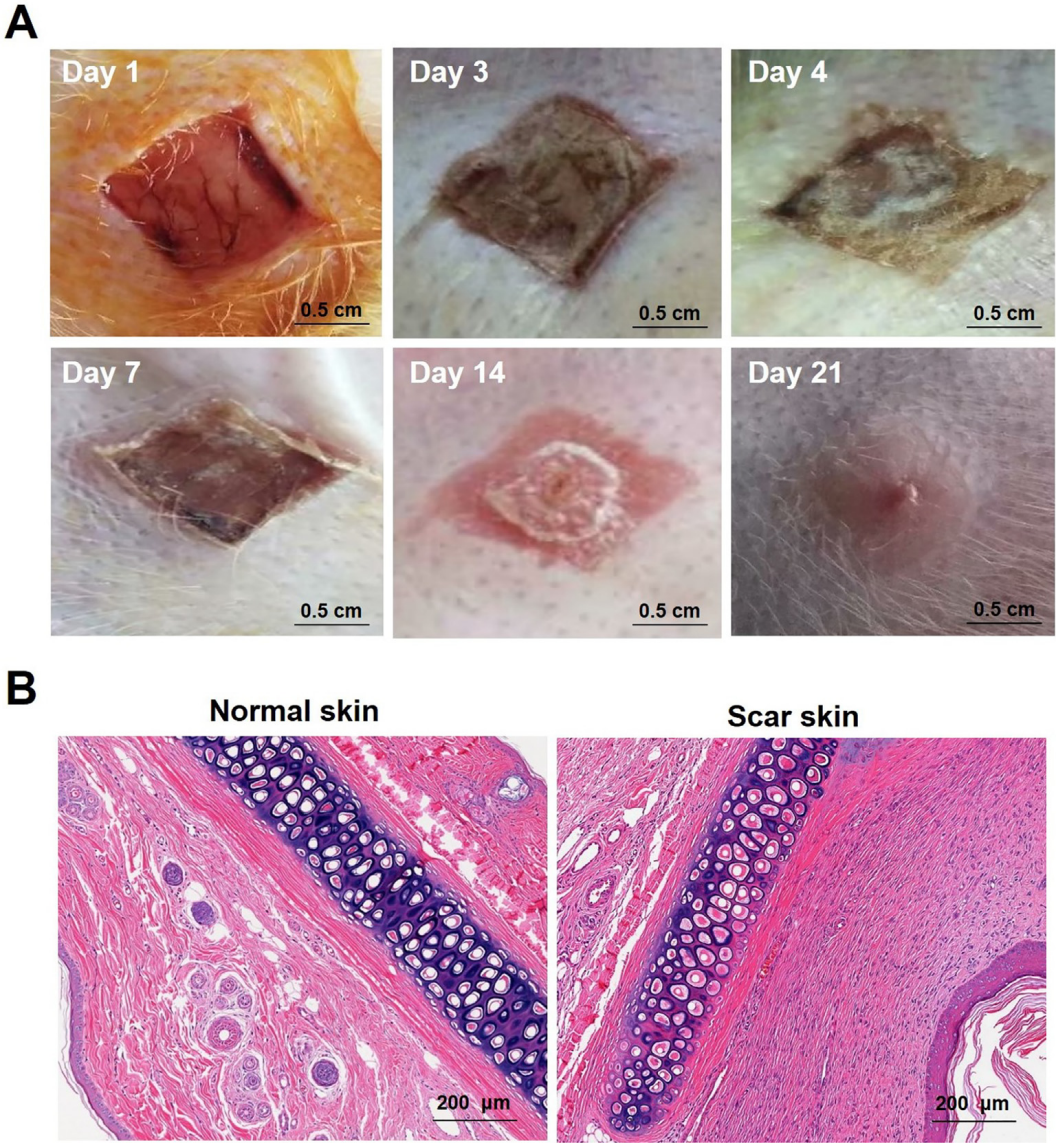
Establishment and evaluation of rabbit ear hypertrophic scar models. Representative photographs for the rabbit ear-based hypertrophic scar models on day 1, 3, 4, 7, 14 and 21 (A). Histologic evaluation of scar tissues in rabbit ear stained with H&E (B).

The rabbit ear-based hypertrophic scar model is a reproducible model for studying scar tissue behavior (Kloeters, Tandara, & Mustoe, 2007). As shown in Fig. 3A, the rabbit ear-based hypertrophic scar models were successfully established by the surgical trauma method. During the entire process of the experiment, the rabbits were in good condition and almost no wound infection happened. The day after surgery, the skin wounds in rabbit ears began to contract. As time went on, scabs were slowly grown and became the hard shells. It was at this time, the hypertrophic scars in rabbit ear were grown and became greater, which ultimately resulted in the fall off of scabs and the appearance of hypertrophic scars and keloids. Compared with the normal skin tissues of rabbit ear, the hypertrophic scar tissue showed obvious uplift and incrassation with a slightly pink tinge and hard texture. Histological analysis showed that a relatively large amount of hypertrophic scar fibroblasts appeared in scar skin, compared with the normal skin, which indicated that the rabbit ear-based hypertrophic scar models were successfully established (Fig. 3B).

### 3.4 *In vivo* anti-hypertrophic scar activity

The rabbits received the experimental treatment by topical smearing on the hypertrophic scar models to evaluate the anti-hypertrophic scar activity of the SCMC ointments. From outside, after treatment with the low, middle and high concentration SCMC ointments, all the rabbit ear scar tissues appeared different degree of shrink and fading, and took a subtle but palpable shift from hard to soft texture. Compared with the treatment groups, the shift of the scar tissues from hard to soft texture didn’t happen in control group. The rabbit ear scar tissues in both groups became light in color compared with normal rabbit ear skin before treatment.

**Fig. 4:**
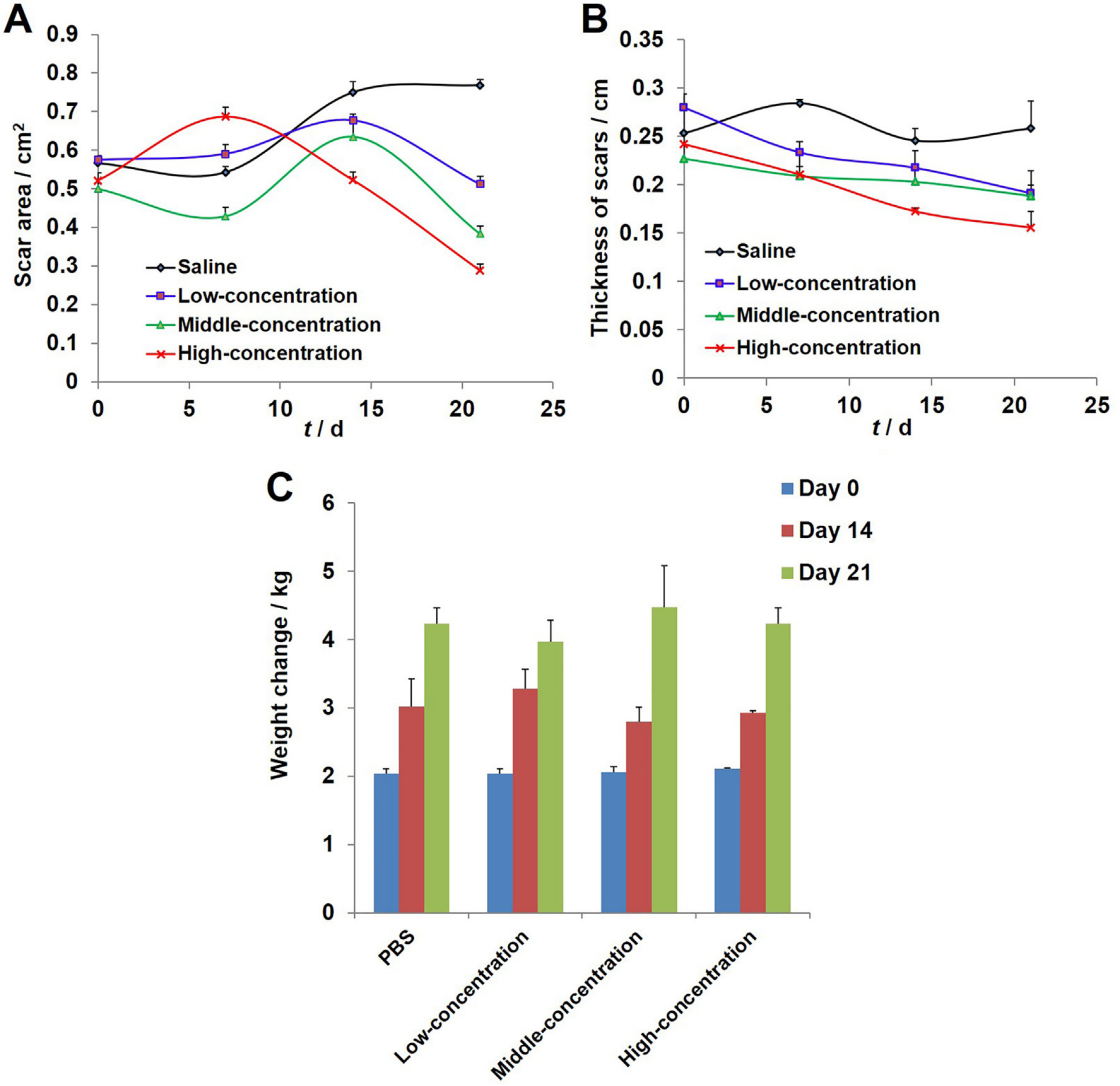
Anti-hypertrophic scar activity (mean ± SD, *n* = 3). (A) Scars area growth curves for rabbit ear-based hypertrophic scars after topical smearing treatment. (B) Scars thickness growth curves for rabbit ear-based hypertrophic scars after topical smearing treatment. The scars were smeared with the SCMC ointments (three times a day). (C) Rabbits body weight changed within 21 d.

In addition, the changes of scars area and thickness were monitored and recorded in detail after the treatment. Fig. 4 A and B presented the hypertrophic scar tissues growth situation after the topical smearing treatment of saline (*n* = 3, 30 mg saline/kg body weight), low concentration SCMC ointment (*n* = 3, 30 mg SCMC ointment/kg body weight), medium concentration SCMC ointment (*n* = 3, 30 mg SCMC ointment/kg body weight) and high concentration SCMC ointment (*n* = 3, 30 mg SCMC ointment/kg body weight). As shown in Fig. 4A and B, the results indicated that the SCMC ointments with the low, middle and high concentration had certain inhibition effect on the hypertrophic scars, and seemed to show a certain degree of concentration dependence to the scar treatment compared with the control group. In all the experimental groups, the high concentration SCMC ointment group exhibited the highest anti-hypertrophic scar activity. For the control group, the hypertrophic scars grew up slowly with a constant speed after 7 d of treatment. The mean scar area and thickness in the low, middle and high concentration groups on day 21 were respectively (0.511 ± 0.10) cm^2^ (*n* = 3, *P* < 0.05) and (0.191 ± 0.06) cm (*n* = 3, no significant), (0.383 ± 0.13) cm^2^ (*n* = 3, *P* < 0.01) and (0.188 ± 0.03) cm (*n* = 3, *P* < 0.05) and 0.288 ± 0.11 cm^2^ (*n* = 3, *P* < 0.01) and (0.155 ± 0.04) cm (*n* = 3, *P* < 0.01), which was significantly reduced when compared with the saline group (0.768 ± 0.10) cm^2^ (*n* = 3) and (0.258 ± 0.07) cm (*n* = 3). After 14 d of treatment, hypertrophic scars in the SCMC ointment group decreased rapidly at a similar rate, and the reduction rate of hypertrophic scars was significantly faster than that in the PBS group. The body weight of rabbits in all experimental groups was increased during the experimental period, and there was no significant difference among these groups (Fig. 4C).

**Fig. 5:**
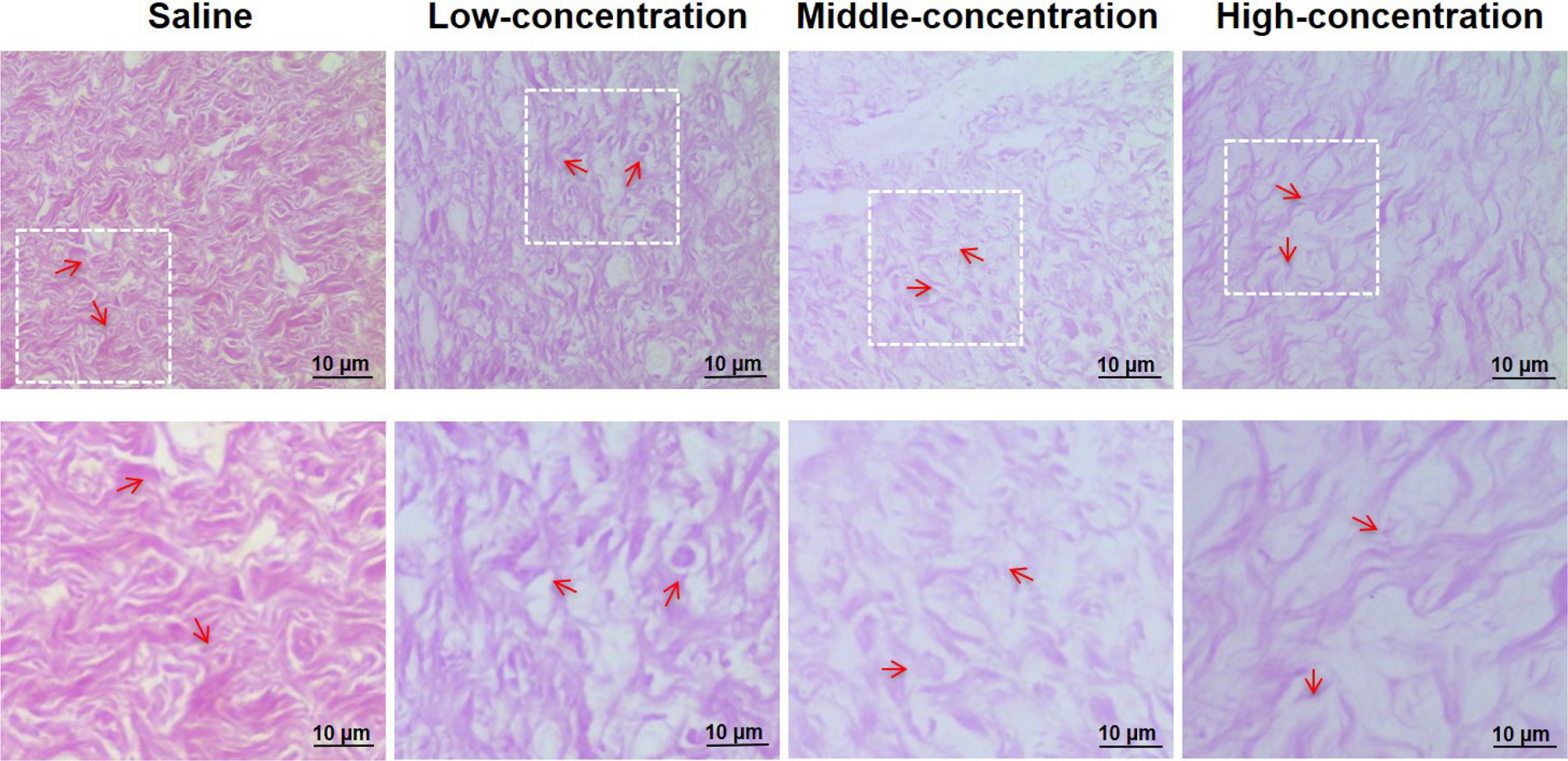
Immunological staining evaluation of hypertrophic scars tissues in rabbit ears treated with each treatment group at 21 d after topical smearing treatment with SCMC ointments and stained with Van Gieson. The second line of figure is photographs at 10× magnification of the square in the first line.

*In vivo* immunohistochemistry results also indicated that the SCMC ointments exhibited good anti-hypertrophic scar effect. Fig. 5 presented the distribution pattern of collagen fibers and reticular fibers of the hypertrophic scar tissues after treatment of 21 d, which were stained with Van Gieson. For the saline group, a significant increase of collagen fiber and reticular fiber distributions was observed clearly under the 40× or higher magnification of the digital microscope, which were arranged closely with small intercellular space. After treatment with the SCMC ointments, the distributions of collagenic fibers and reticular fibers were decreased, and the collagen fibers were arranged loosely, especially in the high concentration group. The improvement degree of the hypertrophic scar was gradually increased along with the treatment concentrations. The results of *in vivo* immunohistochemistry were basically consistent with experimental results in the *in vivo* anti-hypertrophic scar effect studies (Fig. 4).

**Fig. 6:**
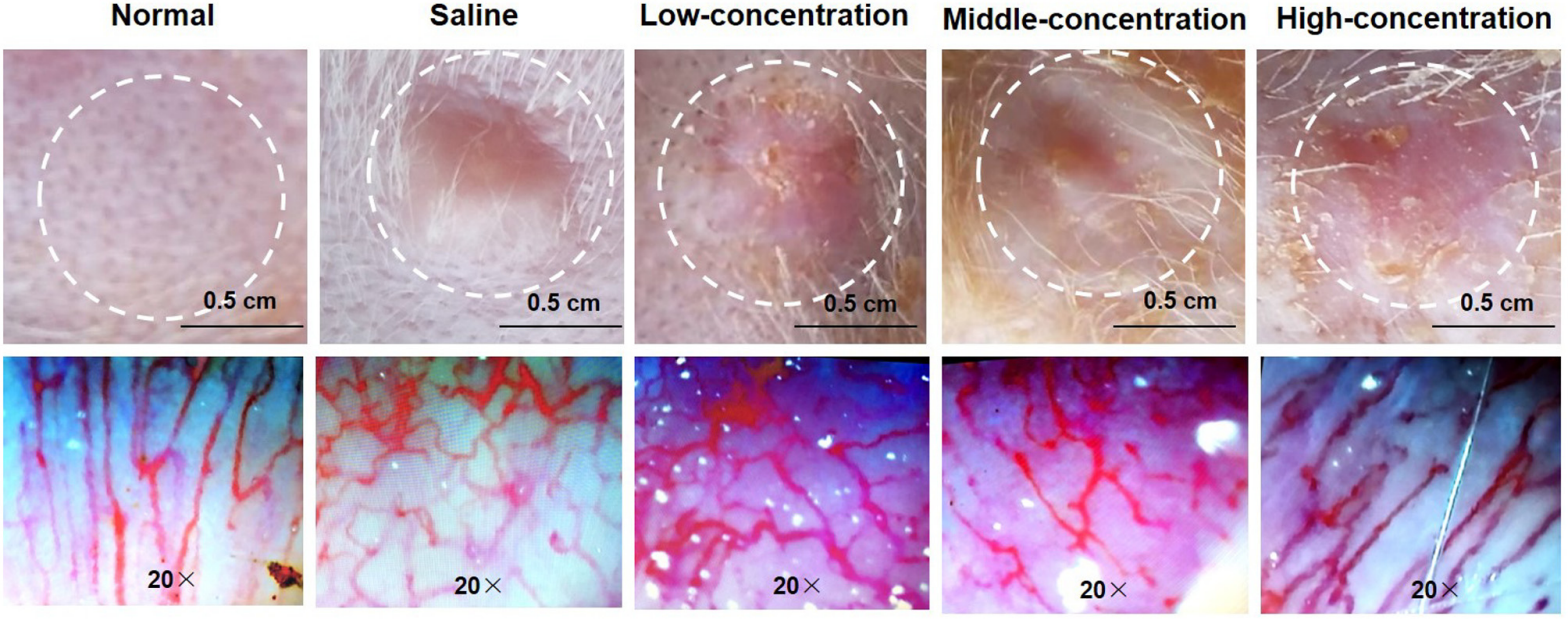
Representative photographs for vascular supply and distribution in rabbit ear-based hypertrophic scar tissues on day 21 after treatment.

Except for the scar area and thickness, the vascular supply and distribution of the hypertrophic scar tissues were also further investigated to evaluate the anti-hypertrophic scar effect of the SCMC ointment, which was observed by the microcirculation blood flow visualize. Fig. 6 showed the vascular supply and distribution of the hypertrophic scar tissues and normal skin tissues. In the normal skin tissues, either large or small vessels were manifested clearly, the blood circulated well and no abnormal hyperplasia of small vessels was observed. In the saline group, the hypertrophic scar tissues experienced the opposite situation compared with the normal skin tissues. The significant increase of local angiogenesis in scar tissue was observed and presented a clustered appearance, uneven distribution of blood vessels and unclear contour. However, after treatment, as shown in Fig. 6, with the increase of drug concentration, the uneven vascular distribution and abnormal vascular increase in scar tissue were alleviated and improved to different degrees. Especially in the high-concentration group, the vascular distribution and supply in scar tissues almost tended to be normal after the topical SCMC ointment treatment.

## 4 Discussion

The problem of skin scarring has conventionally been approached as a pathology of the dermis. Nonetheless there are increasing experimental evidence and clinical data showing that the epidermis plays a major role in scar formation (Mustoe & Gurjala, 2011). The tissue repair and regeneration-regulating mechanism out of control could be the cause of hypertrophic scars and keloids in the wound healing process (Wolfram, Tzankov, Pülzl, & Piza-Katzer, 2009). Peculiarity of hypertrophic scars and keloids is excessive collagen deposition in the wound healing process, which has brought a major therapeutic dilemma and challenge to a broad spectrum of the medical profession. For centuries, hypertrophic scars and keloids have affected patients and frustrated physicians (Alster & Tanzi, 2003). As well, they may result in significant cosmetic disfigurement (Urioste, Arndt, & Dover, 1999).

Ointment is considered as the simplest and convenient medication dosage form with high compliance and wettability (Kandhare et al., 2016), which enjoys a good quality in adhesion, spread stretchability and portability. Because of these advantages, ointment is widely employed as vehicles for transdermal drug or cosmetic delivery and has potential application prospects on the skin-based diseases (Lepselter et al., 2017). This research was based on the theory of traditional Chinese medicine and related principle of “Huo-Xue-Hua-Yu, Ruan-Jian-San-Jie” (Chen and Chen, 2015, Xu et al., 1989), to screen Chinese medicines with potential anti-scarring effect and finally prepared the SCMC ointment for the further treatment of the hypertrophic scars and keloids. Combining the advantages and characteristics of Chinese medicine, a Chinese medicine-based composition (SCMC) ointment containing the extracted active ingredients of six Chinese herbal medicines is recommended as a viable treatment option for scar management in this study (Fig. 1). To ensure quality of SCMC ointment, it is most important that the ointment bases have been further screened and optimized by the orthogonal design and a series of verified experiments. The results showed that the SCMC ointment was successfully prepared by an emulsification method (Fig. 2), which has provided a simple and effective treatment method for hypertrophic scar through topical drug administration onto the scar models surface (Fig. 3).

After three weeks of the SCMC ointments treatment, the results showed that the rabbit ear scar tissues appeared different degrees of shrink and fading, and took a subtle but palpable shift from hard to soft texture in the treatment process (Fig. 5). In addition, the results also indicated that the SCMC ointments seemed to show a certain degree of concentration dependence to the scar treatment compared with the control group, the high concentration SCMC ointment group exhibited highest anti-hypertrophic scar activity in all the experimental groups (Fig. 4A and B). The mean scar area and thickness in the low, middle and high concentration groups on day 21 were significantly reduced when compared with the saline group. This could be due to the releases of the extracted active ingredients of six Chinese herbal medicines into the epidermis and dermis tissues and play a synergistic therapeutic effect on hypertrophic scar. Of course, the therapeutic action of the SCMC ointment in hypertrophic scars was related to the potential anti-hypertrophic scar effect of extracted active ingredients of six Chinese herbal medicines, such as that tanshinone IIA of *S. miltiorrhiza* could downregulate survivin and deactivate Keloid fibroblasts (Chen, Liang, Liang, Li, & Liu, 2016), matrine of *S. flavescens* might obviously enhance the fibroblast apoptosis in rabbit ear hypertrophic scar (Tang et al., 2002).

Interestingly, *in vivo* immunohistochemistry results also indicated that the SCMC ointments had good anti-hypertrophic scar effect (Fig. 5). After treatment with the SCMC ointments, the distributions of collagenic fibers and reticular fibers were decreased, and the collagen fibers were arranged loosely compared with the saline group, especially high concentration group. In addition, with the increase of drug concentration, the uneven vascular distribution and abnormal vascular increase in scar tissue were alleviated and improved to different degrees (Fig. 6). These experimental results showed that the principle of Chinese medicine theory, activating blood and resolving stasis, softening hardness and dissolving lump (i.e. “Huo-Xue-Hua-Yu, Ruan-Jian-San-Jie”) was feasible and had the significant guiding sense to the treatment of hypertrophic scars. We also observed that the body weight of rabbits in all experimental groups increased during the experimental period (Fig. 4C), and there was no significant difference among these groups, which indicated that the SCMC ointment is very safe for the rabbits.

## 5 Conclusion

In this study, we prepared a Chinese medicine composition ointment (i.e. SCMC ointment) under the guidance of Chinese medicine theory, which, according to the final optimal formulation of ointment bases, was composed with stearic acid 17.5%, glycerol monostearate 4% and sodium dodecyl sulfate 1.25%, ethyl4-hydroxybenzoate 0.15%, azone 4%, white vaseline 8% and glycerin 13.6%, and including six Chinese herb active extracts. Our results suggested that all the quality control indications of the SCMC ointment, including appearance, centrifugal stability, heat/cold resistant properties, pH range and skin stimulation met the requirements. This work demonstrated the feasibility of using a Chinese medicine composition ointment to treat hypertrophic scars. *In vivo* anti-hypertrophic scar activity results showed that all the rabbit ear scar tissues appeared different degree of shrink and fading and took a subtle but palpable shift from hard to soft texture after treatment with the low, middle and high concentration SCMC ointments. In addition, the mean scar area and thickness in the low, middle and high concentration SCMC ointment groups on day 21 were significantly reduced compared with the saline group and seemed to show a certain degree of concentration dependence to the scar treatment. *In vivo* immunohistochemistry results also indicated that the SCMC ointments had good anti-hypertrophic scar properties and could inhibit hypertrophic scar formation. More interestingly, the SCMC ointments could improve the blood circulation conditions in the hypertrophic scar tissues. Our study provides a simple and convenient six-herb Chinese medicine composition ointment with good anti-hypertrophic scar properties, which makes it a very promising candidate for the treatment of hypertrophic scars and provides a theoretical basis for further clinical application.

## CRediT authorship contribution statement

**Zu-hua Wang:** Conceptualization, Formal analysis, Funding acquisition, Project administration, Resources, Visualization, Writing - review & editing. **Xue-yan Sun:** Formal analysis. **Jiao-jiao Zhang:** Visualization, Writing - review & editing. **Francesca Giampieri:** Visualization, Writing - review & editing. **Cheng-ju Jiang:** Formal analysis, Project administration. **Ting-ting Feng:** Formal analysis. **Zhi-wei Wang:** Project administration. **Rong-yi Chen:** Formal analysis. **Maurizio Battino:** Conceptualization, Resources, Visualization, Writing - review & editing. **Ying Zhou:** Conceptualization, Funding acquisition, Project administration, Resources.

## Declaration of Competing Interest

The authors declare that they have no known competing financial interests or personal relationships that could have appeared to influence the work reported in this paper.
